# Impact of home-based management on malaria outcome in under-fives presenting in a tertiary health institution in Nigeria

**DOI:** 10.1186/s12936-017-1836-6

**Published:** 2017-05-03

**Authors:** Damian U. Nwaneri, Ayebo E. Sadoh, Michael O. Ibadin

**Affiliations:** Department of Child Health, University of Benin Teaching Hospital, P.M.B. 1111, Benin City, Nigeria

**Keywords:** Home-based, Malaria, Management, Morbidity, Mortality, Severity, Parasitaemia

## Abstract

**Background:**

Home-based management of malaria involves prompt delivery of effective malaria treatment at the community by untrained caregiver. The aim of this study was to document home-based treatment of suspected malaria by non-medical caregivers and to identify its health impact on malaria outcome (severe malaria prevalence, parasite load and mortality) in children (6–59 months).

**Methods:**

A descriptive cross-sectional study carried out from June 2012–July 2013. Data was obtained by researcher-administered questionnaire and malaria was confirmed in each child by microscopy. Analysis was by Statistical Package for Scientific Solutions version 16.

**Results:**

Of the 290 caregivers (31.2 ± 6.1 years)/child (21.3 ± 14.4 months) pairs recruited, 222 (76.6%) caregivers managed malaria at home before presenting their children to hospital. Majority (99.0%) practiced inappropriate home-based malaria treatment. While only 35 (15.8%) caregivers used the recommended artemisinin-based combination therapy, most others used paracetamol either solely or in combination with anti-malarial monotherapy [153 (69.0%)]. There was no significant difference in mean [±] parasites count (2055.71 ± 1655.06/µL) of children who received home-based treatment and those who did not (2405.27 ± 1905.77/µL) (t = 1.02, *p* = 0.31). Prevalence of severe malaria in this study was 111 (38.3%), which was statistically significantly higher in children who received home-based malaria treatment [90.0%] (χ^2^ = 18.4, OR 4.2, *p* = 0.00). The mortality rate was 62 per 1000 and all the children that died received home-based treatment (*p* < 0.001). While low socio-economic class was the significant predictor of prevalence of severe malaria (β = 0.90, OR 2.5, *p* = 0.00), late presentation significantly predicted mortality (β = 1.87, OR 6.5, *p* = 0.02).

**Conclusions:**

The expected benefits of home-based management of malaria in under-fives were undermined by inappropriate treatment practices by the caregivers leading to high incidence of severe malaria and mortality.

## Background

Malaria poses enormous public health burden especially in sub-Saharan Africa where malaria infection rate rises rapidly from zero to 2.0% during the first 3 months of life and reaches 50.0% by the age of 1 year [1]. The rate remains persistently high through the period of childhood [1, 2]. It is a common cause of death especially in children under the age of 5 years. The management of a febrile child by its direct caregiver using medication bought from a untrained patent medicine vendor or other private providers without a definitive diagnosis is a common practice in most communities in sub-Saharan Africa [1, 2]. To reduce the burden of the disease, the World Health Organization (WHO) under the roll back malaria (RBM) initiative recommended home-based management of malaria (HMM) [1, 2]. The HMM strategy has the following elements; provision of platform that enables caregivers recognize malarial illness early and respond appropriately; community-based training programmes that provides caregivers with the knowledge and capacity to respond to malarial illness and creation of environment that enables the strategy to be implemented by making medicines available as near to the home as possible. This is aimed at delivering effective anti-malarial medicines by non-medical caregivers to individuals with suspected malaria as close as possible to their homes and work places [3].

Although, HMM is veritable tool aimed at reduction of malaria burden, its impact on malaria morbidity and mortality in Africa had shown some contradictory results [3–8]. For example, a systematic review of impact of HMM in Africa by Hopkins et al. [4] showed that there was no significant impact of HMM on malaria morbidity and mortality in two separate works in Kenya and The Gambia [5, 6]. Two separate studies in Zaire and Burkina Faso showed decrease in severe malaria morbidity but no impact on mortality [7, 8]. Orimadegun et al. [9] in Ibadan, South-west Nigeria observed that the risk of severe malaria in under-fives who received home-based treatment compared to those who did not but presented to the health facilities in acute disease was 1.63 and mortality was fourfold higher in the same group of children. Of note that is that, most of these researches were carried out during the era when chloroquine was the drug of choice for treatment of uncomplicated malaria.

In 2005, there has been a change in drug treatment policy of uncomplicated malaria from chloroquine to artemisinin-based combination therapy (ACT) due to the increasing development of parasites resistant to chloroquine [10] Since then, there have been community Advocacy, Communication and Social Mobilization (ACSM) on appropriate HMM by the various malaria control parastatals and partners in malaria endemic countries [11, 12]. The efforts had followed multi-pronged approaches across healthcare providers and consumers at all levels of care. The thrust included recognition of symptoms of suspected cases of malaria, in view to commence treatment within 24 h of onset of symptoms using ACT, given at correct dosages and intervals [2, 10–12]. There is a well-organized malaria programme at the community, ward, local government, State and Federal levels in Nigeria. In Benin City, the programme comprised training and re-training of healthcare providers, patent medicine vendors and role model caregivers at every level on current community case management of malaria. There were well documented modules for such trainings for health care providers at secondary and primary levels, modules for role model caregivers, patent medicine vendors (PMV) and community-case management of malaria. There was also mass community awareness on malaria control programmes through community rallies, radio jingles, use of flyers and other information education communication (IEC) materials in English and local dialects in the preceeding 5 years of this study. The community members were taught to recognize symptoms of malaria (suspected malaria), when to commence treatment, use of the recommended treatment options and recognition of dangers signs for hospital care. Although there was no pre-packaged anti-malarial drugs meant for HMM in the study locale but most available recommended anti-malarial drugs in the communities (at the PMV stores) and health facilities were mostly from the Affordable Medicine Facility-malaria (AMF-m) scheme. The ‘Green Leaf’ logo on the packs of each anti-malarial is for easy identification by all in the community. To augment the efforts of the healthcare providers at the primary health care, the State has trained several role model caregivers/mothers; most of whom are on volunteer services. These were saddled with the responsibility of recognition and treatment of cases of suspected malaria at the communities. They also teach and serve as role models for other mothers/caregivers on malaria case management at the communities.

To provide insight into the progress made so far on HMM programme in Nigeria, this study aimed at documenting the non-medical caregivers’ HMM practices and to identify the impact of these practices on outcome (severe malaria prevalence, level of parasitaemia and mortality) in under-fives who presented with malaria in a tertiary health institution in the South-south region. It is envisaged that the findings of this research will serve as basis for targeted health education meant to improve community case management of malaria.

## Research hypothesis

### Null hypothesis

Home-based treatment does not significantly have impact on malaria outcome (severe malaria, level of parasitaemia and mortality) in under-fives.

### Alternate hypothesis

Home-based treatment of malaria significantly have impact on malaria outcome (severe malaria, level of parasitaemia and mortality) in under-fives.

## Methods

This study was carried out in the Children’s Emergency Unit of the University of Benin Teaching Hospital (UBTH), Benin City. The unit comprises a Children Emergency Room (with average bed capacity of 15 and average patient load of 80 per month) and a paediatric casualty room where out-patient paediatric cases are seen daily. UBTH is a tertiary health facility that serves the people of Edo State. The hospital is located within Benin City such that it takes average of 30–45 min-drive from the farthest point radius of the City to the hospital. However, the hospital serves referral centre for people from the neighbouring states in the South-south, South-west and North-central Nigeria. Majority of these States are within the tropical rain forest where malaria transmission is holo-endemic and stable throughout the year [10–12].

The study which was cross-sectional descriptive was carried out between June 2012 and July 2013. The sample size was determined by the formula [n = z^2^pq/d^2^] proposed by Araoye et al. [13] at 95.0% confidence level, statistical power of 80.0%, degree of freedom of 5.0% and *p* = 0.891 being prevalence of malaria home-based treatment practices of caregivers in Edo State, Nigeria [14] Sample size obtained from this formula was the minimum sample size set for this study.

The study participants were (a) apparently well-nourished children (aged 6–59 months) with suspected malaria and their caregivers. Suspected malaria was defined as presence of fever in any child in whom the mother/caregiver thought malaria as the cause of the fever [11, 12]. (b) Each child had received treatment; some of which may have been non-specific treatment like paracetamol given at home by their caregivers/mothers for suspected malaria. (c) Each caregiver recruited had no formal medical education and was the primary caregiver to the child; (c) all eligible children recruited had laboratory confirmation for malaria by microscopy and parasite count estimation using standard protocol [15]. (d) Children whose caregivers did not give any home-based treatment but brought the child to the health facilities for treatment of suspected malaria during the course of the study were identified for comparative analysis and appropriate interpretation of data.

Excluded from the study were (a) any child without laboratory evidence of malaria. (b) Children who had clinical/laboratory evidence of localized infection such as pneumonia, meningitis, sepsis; and (c) children with malnutrition. These diseases are common cause of fever in children and are associated with increased childhood mortality [16]. (d) Children with fever but their caregivers did not think malaria as the cause of the fever whether or not they received any form of home-based treatment. (e) Children of healthcare providers such as doctors, nurses, pharmacists, community health officer or extension workers, etc. because these group of caregivers are also involved in the HMM campaign programme [10–12].

### Data collection and sampling technique

A semi-structured interviewer-administered questionnaire was used to obtain data from each child’s caregiver. The questionnaire was validated by extensive literature review and was pre-tested on 20 caregiver/child pairs who were excluded from the final analysis. Each caregiver/child pair was recruited consecutively until the calculated sample size was met.

Adequate medical history was obtained and full systemic examination performed for each child by the lead researcher. Children with clinical features in keeping with the WHO case definition of severe malaria were classified as severe malaria while children without such features were classified as uncomplicated malaria [1, 2]. All children with severe malaria were admitted in the hospital and received routine in-patient care in accordance with WHO guidelines and National Anti-malarial Treatment Protocol for malaria. Children with uncomplicated malaria received the recommended ACT and were also followed-up on out-patient basis on the 3rd day of commencement of anti-malarial drugs [2, 10]. The family social class of the study participants was determined as described by Olusanya et al. [17] using mother’s level of education and the father’s occupation. In this method of classification of social class, specific scores are allotted to the father’s occupations and educational qualifications of the mother; and the sum of these scores are used to classify the families into social classes I, II, III, IV, and V. Children whose parents had scores of I and II belong to upper social class, score III (middle social class) and scores IV and V (lower social class).

Malaria parasite diagnosis was made following standard protocol [15]. Thick and thin films were used for determining malaria parasite density and species identification respectively. Samples were collected at presentation and sent to the laboratory immediately. Most samples were analysed within 2 h of presentation in the hospital research laboratory by a WHO malaria microscopist. A 100 high power (100× objective) microscope fields for each slide were examined and malaria parasite recorded as positive or negative. Parasite count for each patient was obtained using the formula proposed by Greenwood and Armstrong and as cited by Cheesebrough [15]. This was done by multiplying the average number of parasite trophozoites counted per high power field (100× objective) by 500. This method had been observed to be more accurate and quicker in determining parasite count and has been recommended by WHO in malaria endemic regions [15].

The outcome variables measured in this study included malaria morbidity at presentation (severe malaria prevalence, parasite load/count) and mortality during the acute phase of the illness.

### Data handling and analysis

The data obtained in this study was analysed by the statistical package for social sciences (SPSS) version 16.0 (Chicago, Illinois, USA). Further analysis was done using GraphPad InStat software (GraphPad Software Inc, San Digeo 92130, USA) where applicable.

The children were stratified into regular age brackets (≤12 months and then 12 monthly intervals) while the mothers/caregivers were conveniently divided into age groups of 10 years interval. Households were categorized as small if they contained ≤5 individuals and large if they contained ≥6 individuals [18].

Concerning HMM, the caregivers were categorized into

- Those that practiced HMM which were caregivers who administered medicine at home for the purpose of treatment of suspected malaria in the child [2, 10–12].
- Those who did not practice HMM which were caregivers who gave no drug for suspected malaria until the child presented in the health facility.

The study participants who practiced HMM were then further classified into

i. Appropriate HMM practices: caregivers that (a) commenced HMM within 24 h of onset of suspected malaria [regarded as early HMM]; beyond 24 h is regarded as late HMM. (b) Used the recommended anti-malarial drugs [ACT]. (c) Administered the ACT at the correct doses and interval]; [2, 10–12]
ii. Inappropriate HMM practices: caregivers who failed in any of the three criteria that defined appropriate HMM practices above.

Presentation in health facility was categorized as early presentation (presentation within 2 days or late (presentation after 2 days of onset of symptoms of malaria) [2].

Quantitative variables in the study (such as number of days before presentation, malaria parasite count, number of days on anti-malarial drugs and number of days on admission) were summarized using means and standard deviations or median and range where applicable. Frequency tables and charts were constructed as appropriate. Pearson’s correlation was used to assess the association between mean parasite count and duration of malaria symptoms. The significance of association was tested using Chi square and Fisher’s exact test as appropriate. Such associations included the relationship between HMM and age of the child, family’s social class, household size and level of education of caregivers. The same tools were also used to assess the association between HMM and malaria morbidity/mortality. Associations with p values ≤0.05 were further analysed by logistic regression model to identify factors (HMM practices, family social class and promptness of presentation) that independently influenced the outcome variables (severe malaria and mortality). The analysis showed that the Nagelkerke R^2^ for severe malaria and mortality was 0.211 and 0.267 respectively indicating that the logistic model has no omitted variables if it fails to reject the null hypothesis. The *p* value of the Hosmer–Lemeshow Test which assessed whether or not the observed event rates match the expected event rates in the subgroups of the model population was 0.869 (severe malaria) and 0.169 (mortality) signifying that the specified logistic regression model is fit and reliable. The level of significance of each test was set at p < 0.05 and 95.0% confidence level.

## Results

There were 290 caregiver (mean age 31.2 ± 6.1 years)/child (21.3 ± 14.4 months) pairs recruited for the study. The male children were 162 (55.9%) and 128 (44.1%) were females [male:female = 1.3:1]. Table 1 shows the socio-demographic characteristics of the children and their caregivers. Majority of the caregivers had secondary 124 (42.8%) and majority 123 (42.4%) belonged to the middle social class.

**Table 1:** Socio-demographic characteristics of the study participants

Socio-demographic characteristics	N = 290 (%)
Children	
Gender	
Male	162 (55.9)
Female	128 (44.1)
Age group (months)	
<12	90 (31.0)
12–23	95 (32.8)
24–35	45 (15.5)
36–47	29 (10.0)
48–59	31 (10.7)
Family social class	
Upper	77 (26.6)
Middle	123 (42.4)
Lower	90 (31.0)
Household size	
Small (≤5)	274 (94.5)
Large (≥6)	16 (5.5)
Caregiver	
Type of caregiver	
Mother	283 (97.5)
Grand-mother	5 (1.7)
Father	2 (0.8)
Age group of caregivers (years)	
16–25	42 (14.5)
26–35	189 (65.2)
36–45	54 (18.6)
>45	5 (1.7)
Level of education of caregivers/mothers	
Tertiary	87 (30.0)
Secondary	124 (42.8)
Primary	65 (22.4)
No formal	14 (4.8)

Two hundred and twenty-two (76.6%) caregivers practiced home-based management for malaria while 68 (23.4%) did not. Only three of these 222 (1.0%) practiced appropriate home-based malaria treatment while 219 (99.0%) did not. Concerning inappropriateness of HMM, commencing HMM late was practiced by 124/219 (56.6%) caregivers, use of inappropriate anti-malarial drugs for HMM 187/219 (85.4%) caregivers and administration of HMM drugs at incorrect dosages was by 215/219 (98.2%) caregivers. Table 2 shows drugs mentioned by caregivers for home-based treatment of malaria and those actually used by the caregivers for home treatment. Paracetamol solely or in combination with anti-malarial monotherapy were the most common drugs used by the caregivers in 153 (69.0%) cases. Some of the monotherapies included chloroquine, artesunate, and sulfadoxine + pyrimethamine (SP). Thirty-five (15.8%) caregivers used the recommended ACT; however, three-quarter of these administered the ACT at incorrect dosages.

**Table 2:** Drugs mentioned by caregivers for home treatment of malaria and those actually used for home treatment

Drug name^a^	Mentioned by caregivers n = 290 (%)	Used by caregivers for HMM n = 222 (%)
Chloroquine	198 (68.3)	30 (13.5)
ACT	152 (52.4)	35 (15.8)
Paracetamol solely or with a monotherapy	171 (59.0)	153 (69.0)
Quinine	151 (52.1)	7 (3.2)
Sulfadoxine + pyrimethamine	118 (40.7)	8 (3.6)
Artesunate	110 (38.0)	8 (3.6)
Herbs	78 (26.9)	3 (1.4)
Antibiotics	2 (0.7)	26 (11.7)
Others		
Camoquine	7 (2.4)	2 (0.9)
Halofantrine		

222 caregivers who practiced HMM for their children

*HMM* home management of malaria, *ACT* artemisinin-based combination therapy

^a^Multiple drugs mentioned and administered by the caregivers

The sources of the drugs used for HMM by the caregivers were mostly from the patent medicine vendors (PMV) in [176/222 (79.0%)] cases, 15.0% from clinics during previous illnesses and 6.0% from other sources such as traditional doctors and neighbours.

Table 3 shows the relationship between such factors as family social class, household size, caregivers’ age and level of education, promptness of presentation and HMM practices. Most of the caregivers from the middle social class significantly were less likely to practice HMM when compared with those from lower and upper social classes (χ^2^ = 6.60, *p* = 0.04). Children who presented late to the health facility were statistically significantly more likely to have received home-based malaria treatment (χ^2^ = 22.89, *p* < 0.001).

**Table 3:** Relationship between socio-demographic factors of study participants, promptness of presentation in health facility and home-based management of malaria

Socio-demographic factors	Home based management of malaria		χ^2^	p
	Yes (%)	No (%)		
Family social class				
Upper (n = 77)	63 (81.8)	14 (18.2)	6.60	0.04
Middle (n = 123)	85 (69.1)	38 (30.9)		
Lower (n = 90)	74 (82.2)	16 (17.8)		
Household size				
Small (n = 274)	208 (75.9)	66 (24.1)	1.13	0.29
Large (n = 16)	14 (87.5)	2 (12.5)		
Caregivers’ age (years)				
16–25 (n = 42)	30 (71.4)	12 (28.6)	0.74	0.86
26–35 (n = 189)	146 (77.2)	43 (22.8)		
36–45 (n = 54)	42 (77.8)	12 (22.2)		
>45 (n = 5)	4 (80.0)	1 (20.0)		
Level of education				
Tertiary (n = 87)	63 (72.4)	24 (27.6)	4.59	0.21
Secondary (n = 124)	92 (74.2)	32 (25.8)		
Primary (n = 65)	56 (86.1)	9 (13.9)		
No formal (n = 14)	11 (78.5)	3 (21.5)		
Promptness of presentation				
Late presentation (n = 124)	112 (90.3)	12 (9.7)	22.89	<0.001
Early presentation (n = 166)	110 (66.3)	56 (33.7)		

Mean malaria parasite count of the children was 2239 ± 1811.41 (range 50–10,500) parasites per µL. There was no case of hyperparasitaemia. There was no statistically significant difference in mean malaria parasite count of children who received HMM (2055.71 ± 1655.06/µL) and those who did not receive HMM 2405.27 ± 1905.77/µL (t = 1.02, *p* = 0.31).

One hundred and eleven (38.3%) of the 290 children presented with severe malaria while 179 (61.7%) had uncomplicated malaria. Mean duration of admission was 5.1 ± 2.2 days. Of those who had severe malaria, 90.0% received HMM. Those who received HMM were 4 times more likely to develop severe malaria when compared who did not receive HMM (χ^2^ = 18.4, OR 4.2, *p* < 0.001) (Table 4).

**Table 4:** Relationship between home-based management of malaria by caregivers and outcomes (level of parasitaemia, severe malaria and mortality)

Outcome	HMM	No HMM	χ^2^	OR	*p*
Level of parasitaemia (/µL)	2055.71 ± 1655.06	2405.27 ± 1905.77	−1.02^a^	–	0.31
Morbidity					
Severe malaria (n = 111)	100 (90.0)	11 (10.0)	18.4	4.2	<0.001
Uncomplicated malaria (n = 179)	122 (68.2)	57 (31.8)			
Mortality					
Yes (n = 18)	18 (100.0)	0 (0.0)	*	12.0	<0.001
No (n = 272)	204 (75.0)	68 (25.0)			

*HMM* home-based management of malaria

* Fisher’s exact test (after compressing the zero in the upper row), p = p value

^a^t test

Mortality rate in this study was 18/290 (6.2%), which was 62 per 1000. Table 4 shows that mortality was 12 times more likely to occur in children whose caregivers gave home-based malaria treatment (100.0%) when compared to none (0.0%) observed in children who did not receive home-based treatment but were brought to the health facility (Fisher’s exact test; OR 12.0, *p* < 0.001).

Table 5 showed that late commencement of HMM significantly was associated with both severe malaria (χ^2^ = 4.90, OR 0.5, *p* = 0.03) and malaria mortality (Fisher’s exact: OR 0.1, *p* < 0.00); use of the ‘not-recommended’ anti-malarial drugs for HMM significantly was associated with severe diseases (χ^2^ = 8.36, OR 0.3, *p* < 0.00).

**Table 5:** Relationship between home management of malaria and outcomes

Components of HMM	Severe malaria		Mortality	
	Yes (%)	No (%)	Yes (%)	No (%)
Promptness of HMM				
Early (n = 98)	36 (36.7)	62 (63.7)	2 (2.0)	96 (98.0)
Late (n = 124)	64 (51.6)	60 (48.4)	16 (13.0)	108 (87.0)
	χ^2^ = 4.90, OR 0.5, *p* = 0.03		Fisher’s exact: OR *0.1*, *p* < *0.00*	
Antimalarial drugs used				
Recommended ACT (n = 35)	8 (22.8)	27 (77.2)	1 (2.8)	34 (97.2)
Not-recommended (n = 187)	92 (49.2)	95 (50.8)	17 (9.1)	170 (90.9)
	*χ* ^2^ = *8.26*, OR *0.3*, *p* < *0.001*		Fisher’s exact: OR *0.3*, *p* *=* *0.32*	
Correctness of dosages				
Correct (n = 7)	2 (28.6)	5 (71.4)	1 (6.8)	6 (93.2)
Incorrect (n = 215)	98 (45.6)	117 (54.4)	17 (8.0)	198 (92.0)
	Fisher’s exact: OR *0.5*, *p* *=* *0.46*		Fisher’s exact: OR *2.0*, *p* *=* *0.45*	

*HMM* home-based management of malaria, *OR* odds ratio

Table 6 is a logistic regression model of factors that influenced malaria outcome in the children using family social class, anti-malarial drugs used for HMM and promptness of commencement of HMM and presentation to health facility as independent variables. At 95.0% confidence level, the model showed that children from lower family social class significantly presented with severe malaria and were two and half times more likely to do so than children from middle and upper classes (β = 0.90, OR 2.5, 95 CL = 1.65, 3.67; *p* < 0.001). The odd that children from the lower family social class would die was 60.0% higher than in children from the middle and upper family classes. Children whose caregivers did not use the recommended anti-malarial drugs for HMM significantly presented with severe malaria (β = −0.94, OR 0.4, 95 CL = 0.16, 0.97; *p* = 0.04). The odds ratio of 0.4 implied that severe malaria disease among the children whose caregivers did not use the recommended anti-malarial drugs was 60.0% higher than among children whose caregivers used the recommended ACT. Children who presented late to health facility were over six times more likely to die when compared with those who presented early (β = 1.89, OR 6.6, 95 CL = 1.42, 30.80; *p* = 0.02).

**Table 6:** Logistic regression model analysis of factors (components of home-based management of malaria, family social class and promptness of commencement of HMM and presentation to health facility) on malaria outcome (severe malaria and mortality)

Factors	Severe malaria β (OR) 95 CL; *p* value	Mortality β (OR) 95 CL; *p* value
Family social class (*lower family social class*)	0.90 (2.5) 1.65, 3.67; <0.001	−0.86 (0.4) 0.18, 1.00; 0.05
Promptness of commencement of HMM (*late HMM*)	−0.33 (0.7) 0.39, 1.31; 0.28	1.45 (4.3) 0.91, 20.04; 0.07
Antimalarial drugs for HMM (*use of the ‘not*-*recommended’ antimalarial drugs*	−0.94 (0.4) 0.16, 0.97; 0.04	1.32 (3.8) 0.44, 31.74; 0.23
Promptness of presentation (*late presentation*)	−0.30 (0.7) 0.41, 1.37; 0.34	1.89 (6.6) 1.42, 30.80; 0.02

*OR* odds ratio, *β* measure of how strongly each predictor variable influences the outcome variables, *95 CL* confidence level, *HMM* home-based management of malaria

## Discussion

This study showed that most children (76.6%) with suspected malaria received home-based management before presentation in the hospital. This finding is in keeping with 81.0% observed by Cowan in Ghana [19], and those observed in previous studies (75.8–89.2%) in different geographical locations in Nigeria [14, 20–22].

Benefits of HMM is observed when it is appropriately administered. This involves caregivers’ recognition of symptoms of malaria and commencement of treatment within 24 h of onset of symptoms using the recommended ACT; given at correct doses and intervals. Failure of caregivers in meeting these criteria renders the HMM interventional strategy ineffective and may undermine its anticipated benefits on malaria outcome. In this study, 90.0% of the children who presented with severe malaria, received HMM and were also 4 times more likely to present with severe disease than the children who did not receive HMM. Late commencement of HMM and use of the ‘not-recommended’ anti-malarial drugs by caregivers significantly were associated with severe malaria. Generally all children who died received HMM as against no death in children who did not receive HMM (*p* < 0.001). This finding was similar to that documented by Orimadegun et al. [9] who reported increased incidence of severe malaria and higher mortality in children who received the ‘not recommended’ anti-malarial drugs at home.

Use of the recommended ACT is a major component of HMM strategy [1, 2, 10–12]. Although many of the caregivers in this study mentioned ACT as the recommended anti-malarial drug treatment for uncomplicated malaria, less than 20.0% of them actually used ACT for HMM. This suggests that knowledge alone may not be enough to influence use of ACT as appropriate HMM drug. Possible factors responsible for use of the ‘not recommended’ anti-malarial drugs for HMM included family social class of the caregivers. Studies in Ghana [19, 23] and Nigeria [24] showed that family income influenced health-seeking behaviour as well as ability to purchase the required medications. For example Jimoh et al. [24] in 2007 observed that a household in Nigeria pays average of USD 7.41 per person in the household for a full course of the recommended ACT. This amount may be too much for families from low family social class who may choose alternative cheaper drugs for home treatment; most of which would be purchased preferably from the patent medicine vendors (PMV). Although the PMV were the most common source of over-the-counter drugs in Nigeria, previous studies had shown that their anti-malarial prescriptions especially for paediatric age groups were usually inappropriate and incorrect [25, 26]. In this present study, most families from the low social class procured their HMM drugs from the PMV, and a significant proportion of these drugs were administered incorrectly by the caregivers.

Paracetamol as a monotherapy or in combination with a monotherapy was the most common drug used for HMM in this study. Although the WHO and National Malaria Treatment Policy encourage the administration of antipyretics, such as paracetamol, to ameliorate fever in addition to the recommended ACT for all suspected cases of malaria in children at the community level [2, 10–12], the use of paracetamol only or in combination with a monotherapy is inappropriate. This practice could largely be attributed to the negative influence of media advertisement of various antipyretics. In these adverts, antipyretics are recommended for home treatment of fever with the addendum that ‘the doctor’ should be seen if symptoms do not abate after 3 days. Caregivers thus delay seeking prompt care at the health facilities in the hope that the child would get better since they have administered some form of treatment.

Late presentation at the health facility for prompt care therefore, became a significant factor associated with higher incidence of severe malaria and increased mortality in this study. This finding is similar to that observed in 2007 by Dada et al. [22] where 74.3% of children with severe malaria presented late to the hospital and the majority were found to have received inappropriate anti-malarial drugs at home. Family social class is a major influence on promptness of presentation to health facilities. It could be postulated that caregivers from the lower social class might often consider the cost of hospital treatment in their decision to seeking care. If the funds were not immediately available, they would likely delay hospital care and opt for HMM which in itself is life-saving if carried out appropriately. Delays in seeking appropriate care for malaria in under-fives could be dangerous as the majority of the children die from malaria and its complication within 2–3 days of the onset of symptoms [27]. Another reason for late presentation was the caregiver’s belief that the child had received medication for their illness, therefore, they would perhaps explore other traditional care if the child fails to get better on the orthodox home-based treatment rather than seeking for hospital care.

## Limitation of study

This study was hospital based with its attendant limitations. For example, having access to ACT in the private sector did not seem to significantly improve the outcomes of HMM as correct regimes were not often followed by this group of caregivers. It would have been interesting to know if in the few cases where regimes were followed correctly the child got better since most children treated properly at home with ACT may not have come to the hospital. This may under-estimate the positive impact of HMM on malaria morbidity/mortality.

## Conclusion and recommendation

Majority of the under-fives in this study received inappropriate home-based treatment of malaria. Although HMM is a veritable tool for malaria control, the expected benefits of this strategy in under-five were undermined by late commencement of HMM and the fact that most caregivers did not use the recommended anti-malarial drugs. The HMM campaign programme, therefore, should emphasize on ACT as the recommended anti-malarial drugs for HMM. It should also highlight the need to improve health education on community case management of malaria especially among the lower family classes and intensify the benefits of early presentation to health facility even when a child had commenced malaria treatment at home.

This paper also demonstrates that seeking care in the unqualified or even qualified private sector is a risk factor for progression to severe disease, therefore, caregivers need better education on understanding what is malaria and the appropriate treatment the child should receive. Also the private providers need more training on the proper management options of malaria including diagnosis by a biological test before prescription.

Finally, to reduce mortality from malaria, drastic action needs to be taken to improve the understanding of how to manage malaria both in the private providers such as the PMVs and caregivers. Unless this can be accomplished, it could be postulated that anti-malarials should be only in the hands of qualified and trained providers in an improved and affordable health system for prompt malaria treatment.
